# The crosstalk effect between ferrous and other ions metabolism in ferroptosis for therapy of cancer

**DOI:** 10.3389/fonc.2022.916082

**Published:** 2022-08-12

**Authors:** Kun Ke, Li Li, Chao Lu, Qicong Zhu, Yuanyu Wang, Yiping Mou, Huiju Wang, Weiwei Jin

**Affiliations:** ^1^ General Surgery, Cancer Center, Department of Gastrointestinal and Pancreatic Surgery, Zhejiang Provincial People’s Hospital (Affiliated People’s Hospital, Hangzhou Medical College), Hangzhou, China; ^2^ Key Laboratory of Gastroenterology of Zhejiang Province, Zhejiang Provincial People’s Hospital, People’s Hospital of Hangzhou Medical College, Hangzhou, China; ^3^ Clinical Research Institute, Zhejiang Provincial People’s Hospital, People’s Hospital of Hangzhou Medical College, Hangzhou, China

**Keywords:** ion metabolism, ferroptosis, glutathione metabolism, lipid peroxidation, cancer therapy

## Abstract

Ferroptosis is an iron-dependent cell death process characterized by excessive accumulation of reactive oxygen species and lipid peroxidation. The elucidation of ferroptosis pathways may lead to novel cancer therapies. Current evidence suggests that the mechanism of ferroptosis can be summarized as oxidative stress and antioxidant defense mechanisms. During this process, ferrous ions play a crucial role in cellular oxidation, plasma membrane damage, reactive oxygen species removal imbalance and lipid peroxidation. Although, disregulation of intracellular cations (Fe^2+^, Ca^2+^, Zn^2+^, etc.) and anions (Cl^-^, etc.) have been widely reported to be involved in ferroptosis, their specific regulatory mechanisms have not been established. To further understand the crosstalk effect between ferrous and other ions in ferroptosis, we reviewed the ferroptosis process from the perspective of ions metabolism. In addition, the role of ferrous and other ions in tumor therapy is briefly summarized.

## Introduction

Ferroptosis is an iron-dependent cell death characterized by reactive oxygen species (ROS) accumulation and lipid peroxidation. Different from cuprotosis, apoptosis and necrosis, it does not depend on classical death pathways such as caspase activation (1–3). The process of ferroptosis is a dynamic equilibrium that includes oxidation and antioxidation mechanisms. On the one hand, ROS accumulation and lipid peroxidation are caused by ferrous metabolism imbalance culminating in plasma membrane damage and cell death. On the other hand, a series of antioxidant systems have been established in humans and other organisms, including cystine-GSH-GPX4, CoQ10-FSP1 and GCH1-BH4-DHFR, which can effectively inhibit lipid peroxidation and thus prevent ferroptosis. Accordingly, it is widely thought that targeting the process of iron metabolism and ferroptosis may provide a potential clinical application for anti-tumor therapy (4).

Iron metabolism plays a central role in ferroptosis. Thus, any imbalance regarding its uptake, distribution and output can initiate ferroptosis. It is well-established that complex mechanisms affect iron absorption, storage, and outflow to prevent or promote the occurrence of ferroptosis, and iron has a bidirectional regulatory effect on the occurrence and progression of tumors (5, 6). In addition to ferrous ions, other ions (Ca^2+^, Zn^2+^, Cl^-^, etc.) have also been reported to participate in ferroptosis, but the regulatory network between ferrous and other ions has not been cleared. Calcium (Ca^2+^) influx in extracellular space is closely related to the ferroptosis process, characterized by depletion of the intracellular antioxidant glutathione, increased reactive oxygen species in the cytoplasm and mitochondrial dysfunction (7). Zinc (Zn^2+^) is widely acknowledged as the second most abundant trace element in humans after iron. Zn^2+^ mainly acts on mitochondria, and its mobilization is the prerequisite for triggering irreversible mitochondrial dysfunction (8, 9). Zinc overload can destroy intracellular calcium homeostasis by damaging mitochondrial function and promoting organelle death in coordination with Ca^2+^-induced damage, eventually leading to cell death such as ferroptosis. Moreover, selenium is an important regulator of ferroptosis, and the utilization of selenium by GPX4 is a necessary condition to prevent ferroptosis induced by hydrogen peroxide (10). GPX4 harbors selenocysteine making cells resistant to irreversible excessive oxidation and ferroptosis. Chloride ions (Cl^-^) are well-recognized as the most abundant anion in the body. Interestingly, it has been shown that an imbalance of chloride ions and channels is associated with ferroptosis (11).

In this review, we discuss the core mechanisms of ferroptosis and introduce the regulatory roles of cations (Fe^2+^, Ca^2+^, Zn^2+^, etc.) and anions (Cl^-^, etc.) in ferroptosis. Furthermore, the potential application value of iron and other ions in treating ferroptosis-associated tumors is also discussed.

## Major mechanisms of ferroptosis

**Figure 1:**
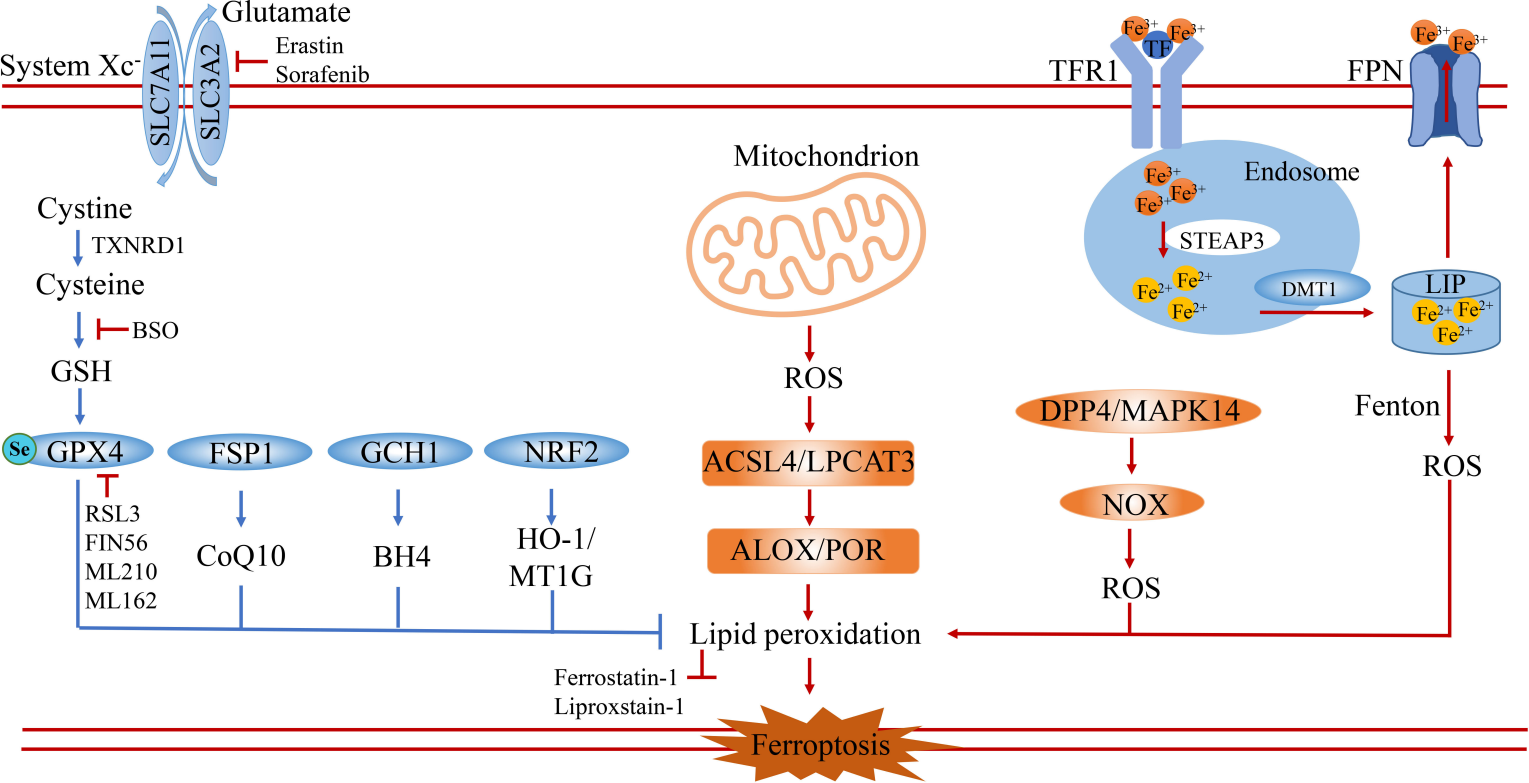
The core signaling pathway of ferroptosis. Ferroptosis is a special type of cell death characterized by excessive accumulation of reactive oxygen species and lipid peroxidation. It is induced by the imbalance of oxidation and anti-oxidation. On the one hand, ferroptosis is induced by the accumulation of ROS from iron-induced Fenton reaction, NOX family and mitochondrion dysfunction. On the other hand, ferroptosis is prevented by anti-oxidation systems including the GSH-GPX4 pathway, FSP1-CoQ10 pathway, GCH1-BH4 pathway and NRF2-HMOX1/MT1G pathway. SLC7A11, solute carrier family 7 member 11; SLC3A2, solute carrier family 3 member 2; TXNRD1, thioredoxin reductase 1; GSH, glutathione; GPX4, glutathione peroxidase 4; FSP1, ferroptosis suppressor protein 1; GCH1, GTP Cyclohydrolase 1; NRF2(NFE2L2), nuclear factor erythroid 2-related factor 2; HO-1, also known as HMOX1, heme oxygenase-1; ACSL4, acyl-CoA synthetase long-chain family member 4; LPCAT3, lysophosphatidylcholine acyltransferase 3; DPP4, dipeptidyl peptidase 4; MAPK14, mitogen-activated protein kinase 14; ALOX, arachidonate lipoxygenase; POR, cytochrome P450 oxidoreductase; TFR1, transferrin receptor 1; TF, transferrin; FPN, ferroportin; DMT1, divalent metal transporter 1; LIP, labile iron pool; BSO, buthionine sulfoximine.

Ferroptosis is a passive cell death process caused by the accumulation of iron-dependent lipid peroxidation and plasma membrane damage. Ever since ferroptosis was defined as a distinct cell death different from necrosis and apoptosis in 2012 by Dixon, vast strides have been made in revealing its main signaling pathways (2), which are mainly categorized into the classical or non-classical signaling pathways. Specifically, the process of ferroptosis is induced by ROS, LOX, and the Fenton reaction, and suppressed by the Cystine-GSH-GPX4 axis and FSP1-COQ10 (**Figure 1**).

### Classic mechanisms

**ROS.** ROS are produced by human and other mammalian cells during cell metabolism, mainly including superoxide (O_2_^-^), hydrogen peroxide (H_2_O_2_), and hydroxyl radical (OH•), which are associated with ferroptosis in redox reactions. The inability to scavenge ROS in time, leads to excessive accumulation of reactive oxygen species, resulting in cell membrane damage and triggering cell ferroptosis. Ferrous ions-mediated Fenton response, mitochondrial ROS, and membrane-associated ROS driven by the NOX protein family are the main sources of intracellular ROS (**Figure 1**). The reaction of Fe^2+^ with H_2_O_2_ is called the Fenton reaction (12). In the Fenton reaction, Fe^2+^ is oxidized to Fe^3+^, and electrons are transferred to H_2_O_2_ to form HO•. The lipid radical (L-OH) binds to O_2_ to form the lipid peroxide radical (L-OO•), which then grabs hydrogen from the adjacent PUFA to produce L-OOH and a new lipid radical. In addition, mitochondria are important sources of intracellular ROS and organelles that regulate ferroptosis (13). Mitochondrial ROS are derived from oxidative respiration in the inner mitochondrial membrane. During electron transfer to O_2_ by mitochondrial electron transport chain complex, part of O_2_ is reduced to O_2_^-^ and H_2_O_2_. Subsequently, O_2_^-^ is rapidly decomposed to hydrogen peroxide (H_2_O_2_) by the superoxide dismutase 2 (SOD2) in the mitochondrial matrix and superoxide dismutase 1 (SOD1) in the membrane gap (14). Another important source of ROS is the membrane-associated ROS driven by the NOX protein family. NADPH oxidase 2 (NOX2) is expressed on the cell membrane and transports electrons through NOX to produce O_2_· or H_2_O_2_ (15). In addition, studies have shown that toxic lipid metabolites produced by lipid peroxidation can trigger NOX expression and form positive feedback to induce ferroptosis in cells (16). The excessive accumulation of ROS is a key factor in lipid peroxidation, leading to ferroptosis in tumor cells.

Interestingly, similar to the mechanism by which tumor cells evade immune cell attack, tumor cells with increased iron and ROS also maintain intracellular ROS balance through complex anti-ferroptosis mechanisms (17, 18).

### Cystine/GSH/GPX4 axis

**System xc^-^**. The cysteine-glutamate antiporter (also known as the System xc^-^) is a complex composed of SLC7A11 and SLC3A2 dimers embedded on the surface of the cell membrane (19). SLC7A11 is the main functional subunit and inhibition of SLC7A11 expression can induce ferroptosis, which has been confirmed *in vivo* by SLC7A11 depletion leading to ferroptosis in pancreatic cancer (20). Erastin, the first identified ferroptosis inducer, acts by inhibiting the activity of the cysteine-glutamate anti-transporter (System xc^-^) (2, 21). In addition to Erastin, drugs such as Salazosulfapyridine (SAS) and Sorafenib have also been shown to be inhibitors of system xc^-^ mediated cystine importation. The system xc^-^ mediates the uptake of cystine during ferroptosis, which is reduced to cysteine by glutathione and/or thioredoxin reductase 1 (TXNRD1) for glutathione biosynthesis. NRF2 (E2-related factor 2) is an important regulator of SLC7A11. Treatment with the drugs such as Erastin, Sorafenib, and Buthionine sulfoximine (BSO) enhances NRF2 activity and prevents ferroptosis (**Figure 1**) **(** 22, 23).

**Cystine/GSH/GPX4 axis.** Glutathione (GSH) is a tripeptide containing an R-amide bond and sulfhydryl. It is composed of glutamic acid, cysteine, and glycine, and exists in two states: reduction (GSH) and oxidation (GSSG). Glutathione peroxidase 4 (GPX4) belongs to the glutathione peroxidase (GPXs) family and is a peroxidase containing selenium. The active center of GPX4 is selenocysteine, which catalyzes GSH to form GSSG. In this process, GSH acts as a reducing agent to reduce toxic lipid peroxides to non-toxic hydroxyl compounds (lipid alcohols), while promoting the decomposition of H_2_O_2_. Inhibition of GPX4 activity (RSL3, FIN56, etc.) leads to the accumulation of lipid peroxides in cells and induces ferroptosis. Current evidence suggests that the Cystine/GSH/GPX4 axis based on the System xc^-^, produces cysteine *via* the sulfur-conversion pathway and selenocysteine through the mevalonate pathway. Therefore, limited cystine input is the initiating factor of ferroptosis. GSH is an essential molecule in GPX4-catalyzed reactions because GSH acts as an electron donor to reduce toxic lipid peroxides (such as P-OOH) to non-toxic lipid alcohols (such as P-OH). In this process, the reduced NADPH acts as an electron donor. Hence, GSH is a key factor in maintaining GPX4 activity and inhibits ferroptosis. On the contrary, treatment with GPX4 active inhibitors such as RSL3 and FIN56 can induce ferroptosis. Indeed, cystine/GSH/GPX4 axis regulation plays an important role in tumor formation. It has been found that SLC7A11 is extensively expressed in various tumors and prevents ferroptosis (2, 24–27). These studies suggest that ferroptosis can modulate the tolerance of tumor cell death.

### CoQ10-FSP1 axis

Coenzyme Q10 (CoQ10) is widely distributed on the cell membrane of mammalian cells and consists of a benzoquinone ring and a polyisoprene tail. Non-mitochondrial CoQ10 reportedly plays an important role as a reversible redox carrier in the electron transport of plasma membrane and Golgi body membrane, which can directly remove lipid peroxy radicals. In addition, oral CoQ10 has been reported to treat various human diseases, such as cardiomyopathy, Parkinson’s disease, and diabetes. In contrast, low CoQ10 levels caused by mutations in CoQ10 biosynthase or related enzymes have been associated with a variety of diseases. The CoQ10 biosynthetic pathway is tightly regulated at both transcription and translation levels. Moreover, CoQ10 can be synthesized by acetyl-CoA through the MVA pathway.

An increasing body of evidence suggests that the sensitivity of GPX4 inhibitors varies greatly between different cell lines. Screening of ferroptosis-related genes using CRISPR/Cas9 revealed that FSP1 is a previously unrecognized ferroptosis suppressor gene (28, 29).FSP1 was initially identified as a p53 response gene (PRG), named PRG3. Due to its homology to human apoptosis-inducing factor (AIF), it is also named mitochondrial-associated apoptosis-inducing factor 2 (AIFM2) (28, 29). Unlike AIF family proteins, FSP1 is predominantly cytoplasmic and may have an affinity for the cytoplasmic surface of the mitochondrial outer membrane. FSP1 is modified by myristoylation and is associated with various cell membrane structures including cytoplasmic membrane, Golgi apparatus, and perinuclear structures. Mutations of the memorial modification site of FSP1 decrease its resistance to ferroptosis. During ferroptosis, FSP1 mainly catalyzes non-mitochondrial CoQ10 to form ubiquinol, and reduced CoQ10H2 or ubiquitin acts as lipophilic free radical to capture antioxidants to prevent lipid peroxides and ultimately block ferroptosis (**Figure 1**) **(** 28, 30). Due to its NADH activity, FSP1 directly reduces lipid free radicals and prevents lipid peroxidation by reducing ubiquinone, or indirectly promotes free radical regeneration of oxidative alpha-tocopherol (a natural antioxidant) to inhibit lipid peroxidation and ferroptosis (31). FIN56, a novel ferroptosis inducer, consumes CoQ10 by modulating the activity of squalene synthase (an enzyme in the mevalonic acid pathway) and enhancing the sensitivity of cells to iron (2, 32).

### GCH1-BH4 axis

GTP cyclic hydrolase 1 (GCH1) has been reported to resist ferroptosis through its metabolites tetrahydrobiopterin (BH4) and dihydrobiopterin (BH2) (**Figure 1**) **(** 33). BH4 can reportedly protect phospholipids containing PUFA tails from oxidative degradation through a dual mechanism. Although the role of GCH1 in protecting tissues and organs from ferroptosis remains to be clarified, deletion of GCH1 in mice has been shown to cause bradycardia and embryonic death in the second trimester of pregnancy. BH4 is an effective free radical capturing antioxidant that reduces lipid peroxidation and inhibits ferroptosis through GPX4. It has been shown that the GTP-dependent cyclic hydrolase 1 (GCH1)/tetrahydrobiopterin (BH4)/phospholipid axis inhibits ferroptosis by synthesizing lipophilic and antioxidant BH4, which enhances cell membrane resistance to oxidative damage and selectively prevents the depletion of phospholipid (34).

### Non-classic mechanisms

GSH-GPX4, FSP1-CoQ10 and GCH1-BH4 are the three main parallel pathways that inhibit ferroptosis, while the NRF2 axis and other antioxidants (such as prominin 2) also play a significant role in suppressing ferroptosis (**Figure 1**). Another endogenous antioxidant defense system is the nuclear factor erythroid 2-associated factor 2 (NRF2), which is usually maintained at low levels by ubiquitination mediated by the tumor suppressor Kelch ECH-associated protein 1 (KEAP1) (35–37). Under oxidative stress, NRF2 is separated from KEAP1 and activated to inhibit ferroptosis through NRF2/SLC7A11/heme oxygenase-1 (HO-1) and NRF2-FTH1 signaling pathways (36, 38). What’s more, NRF2, a transcription factor can regulate ferroptosis in response to oxidative stress through HO-1 and FTH1 and *via* transcription-induced expression of GPX4, SLC7A11, and p53 (35, 39). In terms of tumor drug resistance, NRF2 is one of the core factors leading to drug insensitivity or drug resistance of cancer cells in response to oxidative stress (40). P450 oxidoreductase (POR) and CYB5R1 generate H_2_O_2_ by transferring electrons from NAD(P)H to O_2_, which then reacts with Fe^2+^ to drive lipid peroxidation and promote ferroptosis (41). Haploid cell screening has helped identify acyl-CoA synthase long-chain isomer 4 (ACSL4) as a key factor in sensitizing cells to RSL3-induced ferroptosis. Moreover, Prominin 2 reduces iron levels by promoting iron output and is considered another negative regulator of ferroptosis (42).

## The crosstalk between iron and other ions metabolism in ferroptosis

### Iron and ferroptosis

Iron is an important component of complex proteins such as hemoglobin, myoglobin, heme enzyme and non-heme compounds. It facilitates the transport and exchange of oxygen in the blood and participates in electron transfer and redox reactions (43). Iron homeostasis is achieved by dynamic coordination of iron absorption, utilization, circulation, storage and output. The imbalance of iron content or distribution leads to the accumulation of iron in cells, which leads to the occurrence of iron-dependent cell death (2).

**Figure 2:**
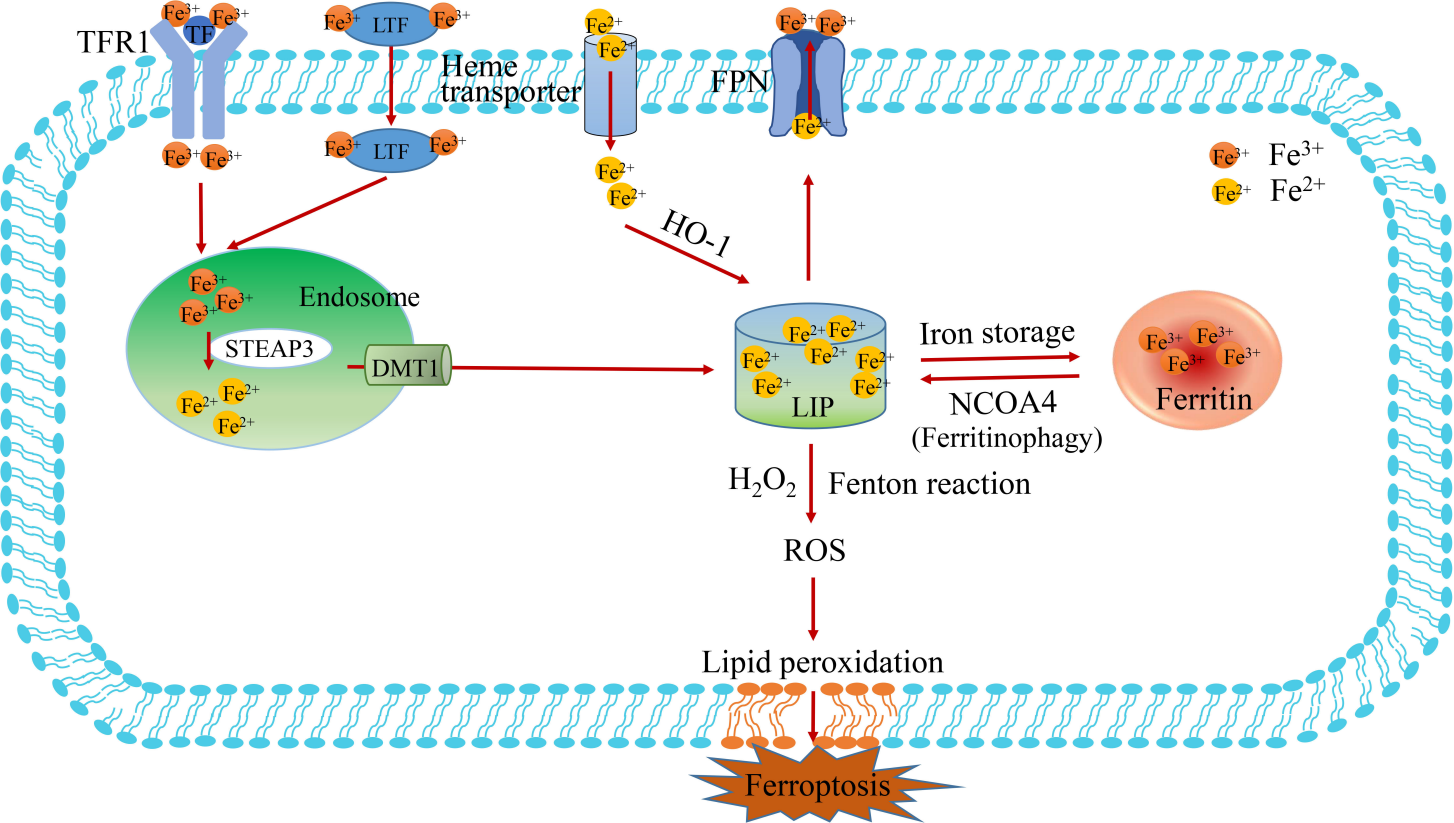
The metabolism of iron. The process of iron import, transduction, storage and export is described in figure 2. The Fe^3+^-loaded transferrin (TF) binds to transferrin receptors1 (TFR1) on the cell membrane to enter the cell. Then, Fe^3+^ is deoxidized to Fe^2+^ by iron oxidoreductase STEAP3 in the endosome, which subsequently Fe^2+^ is released into the labile iron pool (LIP) by DMT1. The pathways of Fe^3+^deoxidization by STEAP3-DMT1, ferritin degradation by NCOA4 (also known as ferritinophagy) and Heme transport are the main sources of Fe^2+^. LIP is the primary storage site for unstable iron, whereas ferritin is the key storage site for stable iron. TF, transferrin; TFR1, transferrin receptor 1; FPN, ferroportin; LIP, labile iron pool; NCOA4, nuclear receptor coactivator 4.

Iron is mostly derived from food absorption and iron recycling in the body. Non-heme iron in food mainly exists as insoluble Fe^3+^ and needs to be reduced to Fe^2+^ until it is absorbed. Iron is transported mainly by transferrin (TF), which is mainly synthesized by the liver and then released into serum globulin, which has a good chelating ability for Fe^3+^ ions (44). Fe^3+^ binds to TF in serum and is then recognized by Transferrin receptor 1(TFR1) on the cell membrane, which delivers the transferrin binding Fe^3+^ into the cell and is located in the endosome (**Figure 2**) **(** 45). In addition to transferrin, lactotransferrin (LTF) participates in the transport of Fe^3+^. Subsequently, Fe^3+^ is released from the transferrin-1 complex in the low-pH acidic endosomes and reduced to Fe^2+^ by six-transmembrane epithelial antigen of the prostate 3 (STEAP3) (6, 46). Finally, the release of Fe^2+^ from endosomes to the labile iron pool (LIP) of the cytoplasm is mediated by divalent metal ion transporter 1 (DMT1, also known as solute carrier family 11 member 2, SLC11A2) (47). Thus, LIP and ferritin represent the two main forms of intracellular iron storage. LIP causes active oxidative stress-related toxicity and is responsible for regulating cellular iron homeostasis through the iron regulatory protein-iron response element system. Ferritin is an iron sequester protein consisting of ferritin light chain (FTL) and Ferritin Heavy Chain 1 (FTH1), containing up to 4500 irons, which can be dissolved by lysosomes or ferritinophagy to release iron ions (48). Ferritin exhibits a variety of functions in iron transport, cell proliferation, angiogenesis and immune suppression, but it is not directly involved in ROS production. Evidence suggests that increased iron in LIP is the main cause of triggering the Fenton reaction and promoting ferroptosis (5).

In terms of utilization, mitoferritin 1 and 2 (MFRN1 and MFRN2) transport iron to the mitochondria to assist cellular respiration and heme synthesis. Ferritin can also release free iron by degradation of nuclear receptor coactivator 4 (NCOA4), a process known as “ferritinophagy”(**Figure 2**). In terms of iron output, ferroportin (FPN, also known as Solute carrier family 40 Member 1, SLC40A1) mediates iron efflux and is the only known protein in mammals that synergistically exports intracellular iron with ceruloplasmin or heparin (49). Ceruloplasmin inhibits ferroptosis by regulating iron homeostasis in HepG2 and Hep3B cells. It has been showed that the consumption of ceruloplasmin leads to intracellular accumulation of iron ions and lipid ROS and promotes ferroptosis induced by erastin or RSL3. Iron regulatory proteins (IRP1/2) are also involved in intracellular iron homeostasis, regulating the expression of genes related to intracellular iron uptake, storage or excretion through post-transcriptional regulation (50). In addition, prominin protein 2 promotes the formation of ferritin poly-vesicles and exosomes that transport iron out of cells, thereby promoting breast cancer resistance to ferroptosis (42).

Excessive iron can produce ROS through the Fenton reaction and activate iron-containing enzymes (such as lipoxygenase) to promote lipid peroxidation and lead to ferroptosis. It is widely thought that complex mechanisms affecting iron absorption, iron storage and iron outflow in tumor cells, to prevent or promote iron death and have a bidirectional effect. A study demonstrated higher iron content in tumor cells than in normal cells, and found that tumor cells promote cell proliferation by increasing iron content (51). Ferroportin (FPN) is the most important iron exporting protein, and FPN exhibits significantly lower expression in tumor cells (49, 52). Overwhelming evidence substantiates that excessive dietary iron or direct iron injection can produce excessive reactive oxygen species (ROS), increasing the risk of solid tumors (53, 54). Moreover, iron imbalance in cells can lead to the development of cancer or the initiation of cell death processes. In addition to causing ferroptosis, iron-mediated ROS may also lead to different types of cell death, such as apoptosis and necrosis. Unfortunately, there is no clinical evidence that supplementing exogenous iron or regulating the intracellular distribution abundance of iron can be used to treat tumors.

### The crosstalk between Ca^2+^ and Fe^2+^-induced ferroptosis

**Figure 3:**
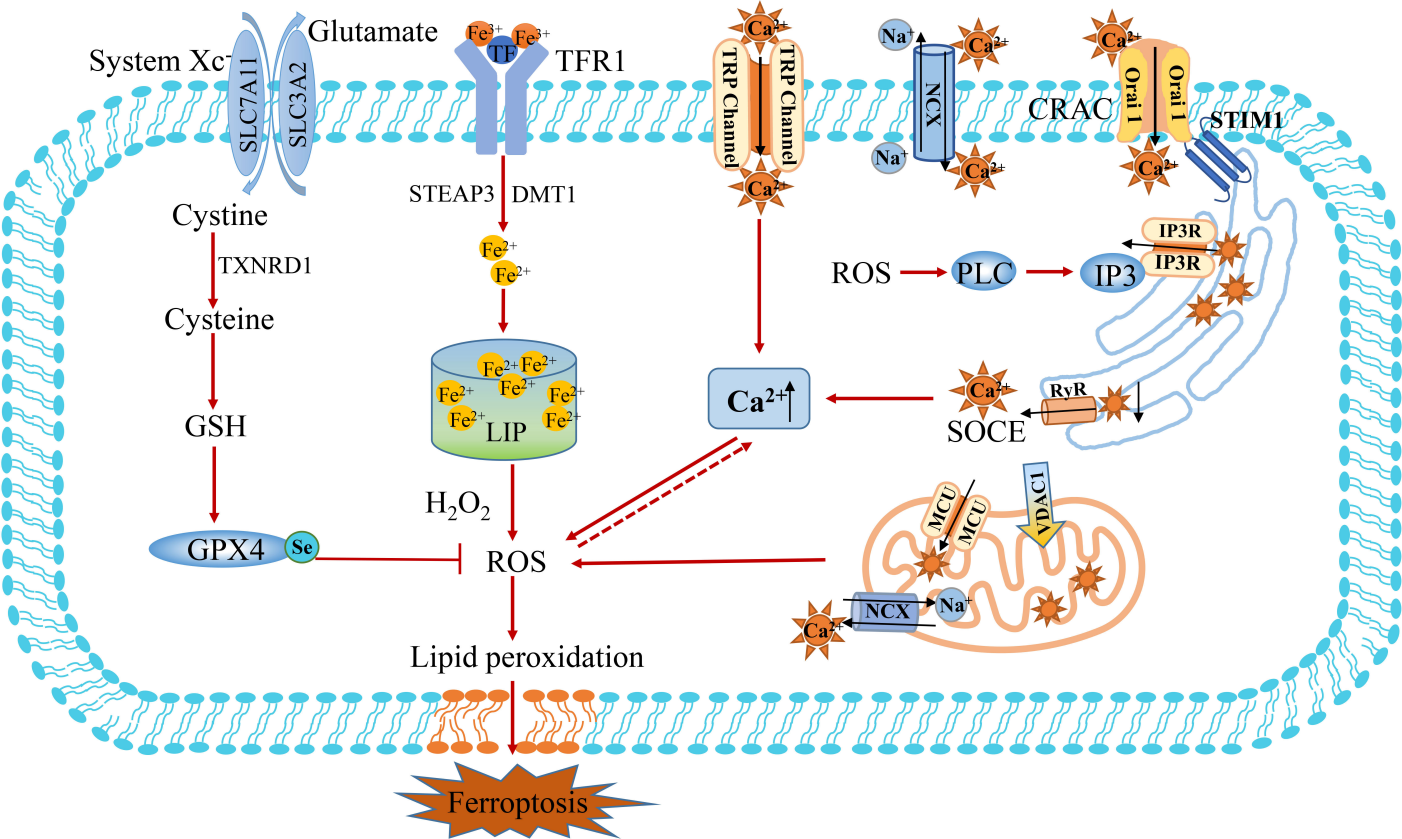
The crosstalk between Ca^2+^ and Fe^2+^ metabolism. Ca^2+^ entry into the cytoplasm depends on the influx of external calcium (Ca^2+^ channels of TRP, calcium pumps, Na^+^/Ca^2+^ exchanger of MCX) and release of internal calcium (IP3 and Ryonadine pathways). The endoplasmic reticulum (ER) binds to Orai1 through calcium receptor STIM1 and mediates the SOCE mechanism to promote calcium influx. Mitochondria participate in cellular Ca^2+^ signaling through their transporters, such as mitochondrial calcium uniporter (MCU) transport cytoplasmic calcium to mitochondria, while mitochondrial sodium-calcium exchangers (NCX) transfer calcium out of mitochondria. The influx of extracellular Ca^2+^can induces the opening of the permeability pore of the mitochondrial bilayer membrane, resulting in mitochondrial swelling and rupture. The release of Ca^2+^ from the mitochondrial intermembrane calcium pool can overload the cytoplasmic Ca^2+^ and induce ferroptosis resulting from the accumulation of ROS. MCU, mitochondrial calcium uniporter; NCX, sodium-calcium exchanger; SOCE, store-operated Ca^2+^ entry; STIM, stromal interaction molecule; TRP, transient receptor potential; VDAC1, voltage-dependent anionic channel 1; CRAC, calcium released activated Ca^2+^ channel.

Calcium (Ca^2+^) is an important second messenger in eukaryotic cells, which affects the changes in intracellular signaling pathways and regulates many different cellular processes (55). Cells generally maintain low cytoplasmic free Ca^2+^ levels under physiological conditions. In this respect, calcium is strictly regulated intracellularly, and the disruption of calcium homeostasis can lead to cell damage and even cell death (56). The distribution of calcium is not uniform, of which 50% is stored in the nucleus, 30% in the mitochondria, 14% in the endoplasmic reticulum, and the rest in the plasma membrane and solute. Calcium is usually transported by calcium ion channels, calcium pumps and other calcium transporters. Ca^2+^ entry into the cytoplasm depends on external calcium influx (Ca^2+^ channels, calcium pumps, Na^+^/Ca^2+^ exchangers) and internal calcium release (IP3 and Ryonadine pathways) (31, 57). The endoplasmic reticulum (ER) is an intracellular Ca^2+^ storage organelle that plays an important role in many cellular metabolic processes. When calcium ions stored in the ER are released *via* IP3R, it can induce extracellular calcium ions to rapidly enter the cell, a process called store-operated Ca^2+^ entry (SOCE) (58). SOCE is mediated by the calcium receptor stromal interaction molecule (STIM) on ER and Orai on the cell membrane (**Figure 3**) **(** 59). The calcium receptor STIM1 on ER moves to the plasma membrane and then binds to Orai1, resulting in the entry of Ca^2+^ from extracellular to intracellular, which regulates various death pathways (60–62). In addition, mitochondria can participate in cellular Ca^2+^ signaling through their transporters, such as mitochondrial calcium uniporter (MCU), which transport cytoplasmic calcium to mitochondria, and mitochondrial sodium-calcium exchange (NCX), which transfer calcium out of mitochondria (63, 64). The regulation of NCX activity can also inhibit Ca^2+^ efflux, promote intracellular Ca^2+^ accumulation by Na^+^ influx, and activate nicotinamide adenine dinucleotide phosphate (NADPH) oxidase to produce superoxide. On the contrary, the outflow of Ca^2+^ mainly depends on the cytoplasmic membrane, the endoplasmic reticulum calcium pump and Na^+^/Ca^2+^ exchangers. Moreover, SARCO/ER calcium ATPase (Ca-ATPase, SERCA) has been proved to be a regulator that restores ER calcium homeostasis by transporting Ca^2+^ from the cytoplasm back to SR/ER.

**Oxidative glutamate toxicity of Ca^2+^.** Disrupted calcium homeostasis can lead to cell death, such as ferroptosis. Ca^2+^ influx in the extracellular space occurs during the late stages of the cascade characterized by depletion of the intracellular antioxidant glutathione, production of reactive oxygen species in the cytoplasm and mitochondrial dysfunction (65). As early as 1989, Murphy et al. reported that inhibition of cystine input through the cystine/glutamate anti-transporter system xc¯ in neuroblastoma X retinal cell line would lead to consumption of antioxidant glutathione (GSH) and Ca^2+^ dependent cell death (66). This type of cytotoxicity has been named oxidative glutamate toxicity or oxytosis, and recently this mode of death has been defined by most researchers as ferroptosis (67). Two reasons account for the conclusion that the process is similar to ferroptosis and that oxidative glutamate toxicity can be inhibited by iron chelating agents. In addition, it has been shown that the ferroptosis inhibitor ferrostatin-1 could inhibit oxidative glutamate toxicity (68). Maher et al. found that compounds can inhibit oxidative glutamate toxicity by blocking mitochondrial ROS production or reducing Ca^2+^ influx to protect cells from erastin or sulfapyridine-induced cell death (2, 67). Mouse hippocampal cell HT22 represents a good model for studying the effects of endogenous oxidative stress. After the extracellular addition of glutamate to inhibit the system xc^-^, glutathione levels decreased in a time-dependent manner (69). Levels of reactive oxygen species (ROS) increased exponentially, triggering surge activation of the signaling pathway, leading to an influx of cGMP-dependent Ca^2+^ and cell death (70). Indeed, it is widely acknowledged that Ca^2+^ influx is essential for cell death. Glutamate-treated HT22 cells did not die when cells were cultured in Ca^2+^-free medium or calcium antagonists blocking Ca^2+^ influx (66, 71). To elucidate the mechanism of Ca^2+^-induced glutamate oxidative toxicity, the researcher analyzed the calcium-sensitive status in glutamate-resistant HT22 cells and found that the difference in sensitivity was due to the downregulation of Ca^2+^ channel ORAI1 rather than the downregulation of Ca^2+^ sensor STIM1 or STIM2. This regulation resulted in a significant reduction in store-operated Ca^2+^ entry (SOCE) (72).

### The crosstalk between Zn^2+^ and Fe^2+^-induced ferroptosis

**Figure 4:**
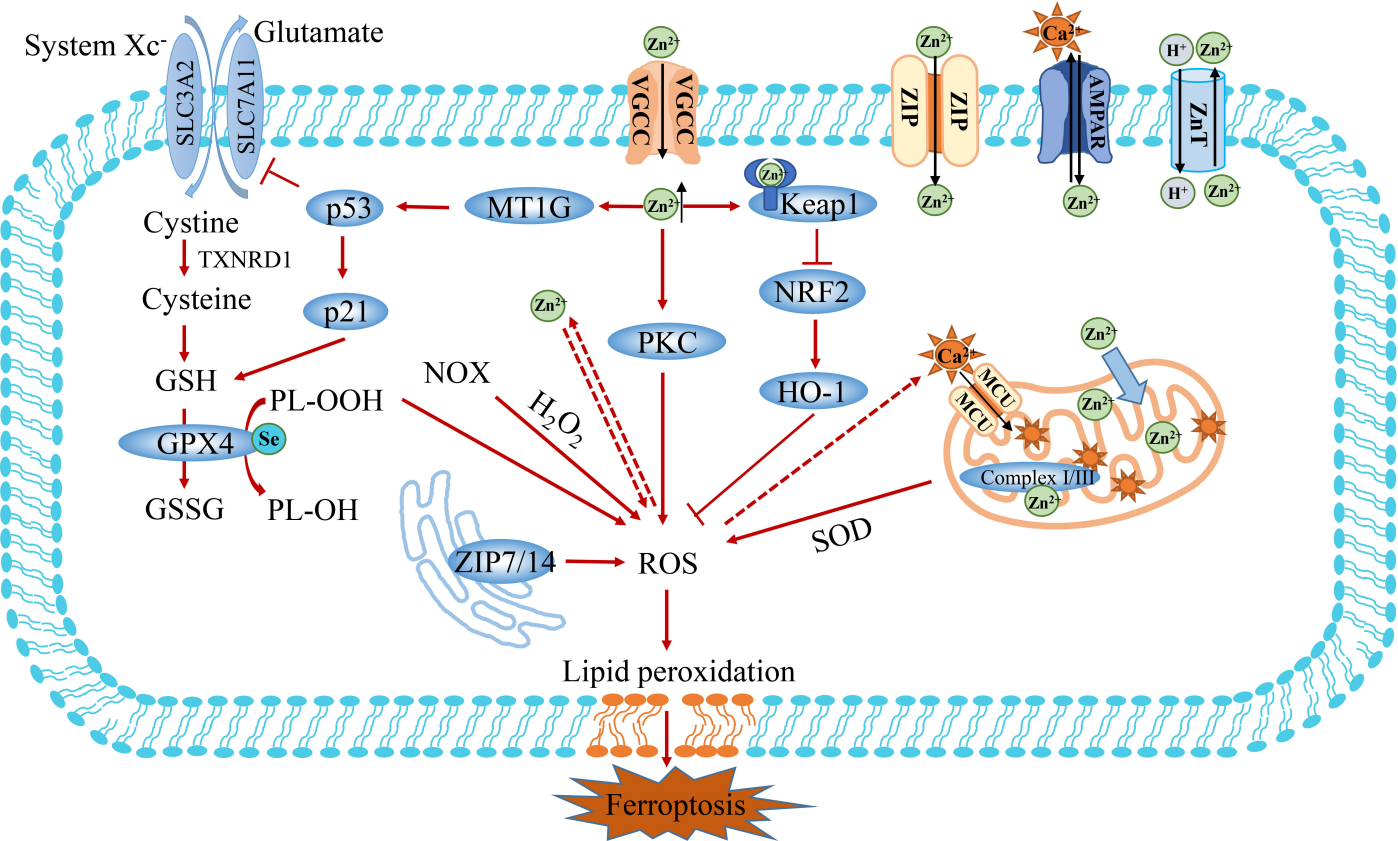
The crosstalk between Zn^2+^ and Fe^2+^ metabolism. Zn^2+^ is transported into cells *via* Zn^2+^-importing proteins (ZIPs), Volage-gated Ca^2+^ channels (VGCCs) and Ca^2+^ and Zn^2+^ permeable GluR2-deficient AMPA channels. Zn^2+^ exposure has been shown to increase the production of ROS and inhibit the activity of GSH. Furthermore, Zn^2+^ can indirectly promote the accumulation of ROS through PKC or Keap1/NFR2 pathways. In addition, the overload of Zn^2+^ induces ferroptosis by activating MT1G/p53 to regulate the expression of SLC7A11 and GSH. Finally, the mitochondrion is an important organelle for the action of Zn^2+^, which affects ROS production by regulating Complex I/III. Keap1, Kelch-like ECH-associated protein 1; FTH1, ferritin heavy chain 1; MT1G, metallothionein 1G; PKC, protein kinase C.

Zinc (Zn^2+^) is the second-largest trace element in organisms after iron, playing an important role in regulating cell metabolism, signal transduction, gene expression and cell death (73). Zinc ions can exist in the form of structural stability and instability. Structural Zn^2+^ is an important cofactor in more than 300 cellular enzymes. It affects organelle functions by binding to enzymes and regulatory proteins as catalytic subunits, such as zinc finger proteins and copper-zinc superoxide dismutase. Zn^2+^-Importing proteins (ZIPs) promote influx of Zn^2+^ into the cytoplasm through activated voltaic gated Ca^2+^ channels (VGCCs) or into neurons *via* Ca^2+^ and Zn^2+^ permeable GluR2-lacking AMPA receptors (**Figure 4**) **(** 74). In contrast, ZnT transporters are homodimers that specifically exchange Zn^2+^/H^+^, removing Zn^2+^ from the cytoplasm (75). Proton-coupled divalent metal ion transporter (DMT1, also known as NRAMP2 and SLC11A2) may be a candidate for Zn^2+^ efflux from lysosomes. DMT1 is an important iron ion transporter, which has been described previously (76). Part of the labile Zn^2+^ in cells is stored in organelles, such as glutamate neurons, synaptic vesicles, mitochondria, lysosomes, endoplasmic reticulum (ER) and Golgi apparatus, and the other part binds to metallothionein (MTs) to form a stable structure (77). MT is a small family of cysteine-rich proteins capable of binding up to seven zinc atoms. Similar to iron, Zn^2+^ can be released from the unstable pool and affect the function of mitochondrial and lysosomes, which participated in the ferroptosis banquet (78). As signaling molecules, Zn^2+^ and Ca^2+^ share many transport and signaling pathways (79). Therefore, Ca^2+^ and Zn^2+^ are synergistically regulated oxidative stress to participate in Fe^2+^-induced oxidative stress and ferroptosis signal.

Overwhelming evidence substantiates that zinc is not only an antioxidant and an oxidative stress sensor, but also a redox inert metal that does not react directly with oxidants (80–82). Zn^2+^ exposure has been shown to increase intracellular ROS production levels. Nonetheless, it remains unclear how Zn^2+^ induces ROS production. It has been established that NADPH oxidoreductase (NOX) and mitochondria are key targets of oxidative stress induced by Zn^2+^(**Figure 4**) **(** 83). Zn^2+^ promotes oxidative stress by activating NADPH oxidase and protein kinase C (PKC) (84, 85). Moreover, Zn^2+^ also acts on mitochondria, and its mobilization is the prerequisite for triggering irreversible mitochondrial dysfunction. The overload of Zinc can disrupt intracellular calcium homeostasis by destabilizing mitochondrial function and promoting organelle death in coordination with Ca^2+^-induced injury, eventually leading to cell death (86–88). Zn^2+^ and Ca^2+^ share many transport and signaling pathways. However, the specific molecular mechanisms underlying the regulatory effect of zinc on calcium are poorly understood.

Cu/Zn superoxide dismutase (SOD1) is an important reactive oxygen scavenging protein, that fights against reactive oxygen-mediated oxidative stress by catalyzing the conversion of superoxide anion to hydrogen peroxide (89). Excess H_2_O_2_ is usually reduced to water by glutathione peroxidase or catalase, preventing the production of hydroxyl radicals. Growing evidence suggests that Zn^2+^ can alter intracellular glutathione content and GSH/GSSG ratio in many cell types (90, 91). Importantly, zinc has been shown to inhibit the activity of enzymes related to glutathione metabolism, especially glutathione reductase (GR), and affect the clearance of endogenous peroxides. Studies have shown that zinc can reduce GSH in astrocytes and increase the output of GSSG to slow down the clearance of exogenous H_2_O_2_, thus promoting the production of intracellular ROS (90). A study showed that zinc could decrease GSSG reductase activity in lung fibroblasts and alveolar cell lines to promote cell death (92). Studies in humans and animals have shown that iron and zinc are negatively correlated and complement each other in intestinal absorption (93–95). Zinc may prevent iron-induced oxidative damage in rats by regulating intracellular iron signaling pathways (96). Zn^2+^ can enhance the expression of Metallothionein 1G (MT1G) in colon cancer cells and enhance cytotoxicity by activating the p53 pathway to promote the sensitivity of cancer cells to oxaliplatin (OXA) and 5-Fu (**Figure 4**) **(** 97, 98). What’s more, Zn^2+^ can increase the expression of heme oxygenase-1 (HO-1) mRNA and protein in colorectal cancer cell lines, which can be inhibited by the iron-binding agent deferoxamine (DFO) (99). Considering the effect of Zn^2+^ exposure on HO-1 expression, the recently documented presence of a conserved Zn^2+^ binding site on the NRF2 inhibitor Keap1 seems to validate the association with Fe^2+^ metabolism (100). Moreover, zinc overload may lead to morphological changes induced by mitochondrial stress, leading to Hela cell death, which was inhibited by the zinc chelating agent TPEN (101). ZIP14 is a transport protein that can mediate cellular uptake of iron, and zinc (102). Increased zinc levers and ZIP14 were observed in the muscle of patients with pancreatic cancer cachexia, it suggested that zinc chelating agents might potentially improve quality of life and prolong treatment survival in patients with PDAC (103). Interestingly, ZnO NPs nanomaterials based on Zn^2+^ can interfere with iron metabolism and induce ferroptosis by regulating iron intake, storage and export. This finding confirms that both ZnO nanoparticles and Zn^2+^ can induce ferroptosis (8). Therefore, zinc supplementation provides a potential therapeutic strategy to sensitize tumor therapy and induce tumor cell death.

### Selenium metabolism and ferroptosis

The trace element selenium (Se) was discovered by Jons Jacob Berzelius in 1817 (104). Later, it was found that selenium in the human body mainly exists in the form of selenoproteins, with 25 kinds divided into two categories. There are 16 kinds of free selenoproteins in cells, the rest are located on the cell membrane, called membrane selenoproteins. Selenoproteins are rare proteins among all kingdoms of life and contain the 21^st^ amino acid, selenocysteine. Selenocysteine is similar to cysteine, except that selenium is substituted for sulfur (105). Albeit the synthesis of selenoprotein is complicated, but it is of great significance to the life activities of humans and other mammals (106).

**Figure 5:**
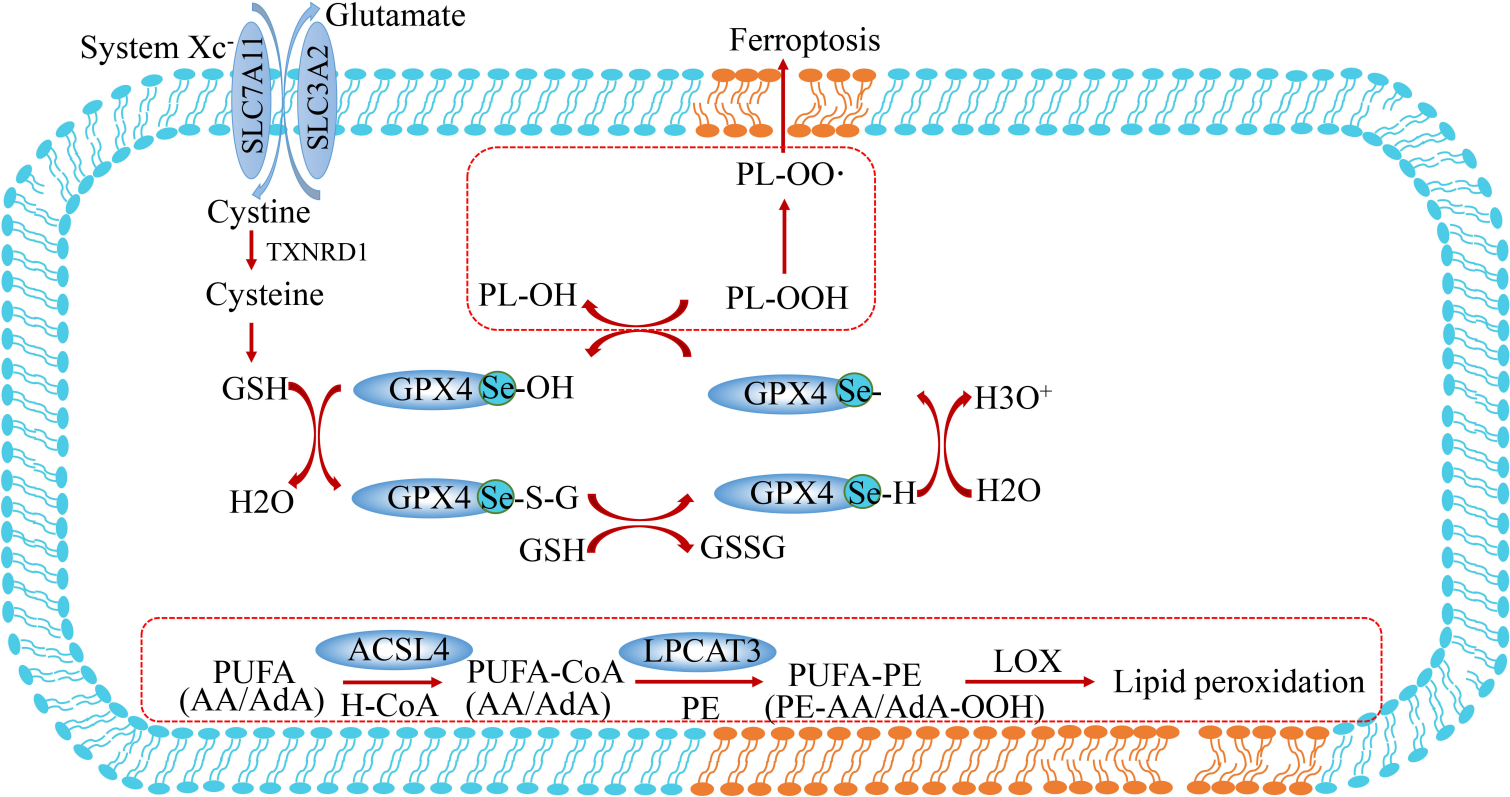
Lipid peroxidation and selenium metabolism in ferroptosis. These peroxides are produced when polyunsaturated fatty acids (PUFA) form PUFA-OOH catalyzed by Acyl-coenzyme A(CoA) synthetase-4 (ASCL4) and LPCAT3. GPX4 converts L-OOH into lipid alcohol (L-OH) by consuming two glutathione (GSH) molecules. PUFA, polyunsaturated fatty acid; PUFA-CoA, PUFA-coenzyme A; FSP1, ferroptosis suppressor protein 1; ACSL4, acyl-CoA synthetase long-chain family member 4; LPCAT3, lysophosphatidylcholine acyltransferase 3.

Studies on selenium-deficient mouse models demonstrated a close association with glutathione peroxidase 4 (GPX4) (107). GPX4 is one of 25 selenoproteins in the human body, and its expression is regulated by selenium content in cells. A study demonstrated that GPX4-deficient mice exhibited embryonic lethality and died at 7.5 days of the embryonic stage. GPX4 harbors selenocysteine making cells resistant to irreversible excessive oxidation and preventing cell ferroptosis (10). GPX4 contains sulfoxide form (GPX-SeOH) and reduction form (GPX-SeH). Under physiological conditions, oxidized GPX-SeOH can be reduced by 2 molecules of GSH to form GPX4-SHE. When GSH depletion or peroxidation occurs in cells, the accumulation of PLOOH leads to ferroptosis (**Figure 5**) **(** 108). Selenium supplementation effectively inhibits GPX4-dependent ferroptosis and inhibits cell death induced by neuroexcitatory toxicity or ER stress in a GPX4-independent manner (109).

The catalytic action of GPX4 is indispensable for normal embryogenesis in mammals, while constitutive deletion of GPX4 has a similar effect to Trsp knockout (110). Moreover, selenium is an important regulator of ferroptosis. The utilization of selenium by GPX4 is essential to prevent ferroptosis induced by hydrogen peroxide. The effect of GPX4 on selenocysteine makes cells resistant to excessive oxidation and ferroptosis (**Figure 5**). SEPHS2 enzymes in the selenocysteine biosynthesis pathway participate in selenium detoxification in cancer cells, which is essential for the survival of cancer cells, highlighting the role of selenium metabolism in cancer. This research suggests that toxic metabolite produced in this process may be a potential direction for cancer treatment (111). In contrast, selenium deficiency has been proved to increase the incidence of many cancers (112). In several clinical trials, oral administration of 200 ug Se per day significantly reduced the incidence of lung, colorectal, and prostate cancers. In this respect, combining sulforaphane and selenium in food leads to synergistic upregulation of TrxR-1, which contributes to enhanced protection against free radical-mediated oxidative damage in human hepatocytes (113). The established role of selenium and GPX4 in anti-oxidation and prevention of ferroptosis suggests they may have potential value for application against the occurrence and prevention of tumors.

### The crosstalk between Cl^-^ and Fe^2+^-induced ferroptosis

Chloride ions are the most abundant anions in the human body. Apart from participating in general physiological functions (regulating cell membrane potential, PH and cell volume, etc.), chloride ions can also act as intracellular signal effectors or second messengers to widely participate in the occurrence and development of diseases and tumors (114–116). There are four types of chloride channels in eukaryotes, including voltage-gated chloride channels (CLCs), ligand-gated chloride channels, and cystic fibrosis transmembrane conductance regulatory channels (CFTR) and calcium-activated chloride channels (CaCCs). Under normal circumstances, immune cells such as neutrophils maintain high levels of cellular chloride ion concentration, which is conducive to the phagocytosis of phagocytes. For example, Cl^-^/H^+^ antiporter and CFTR transporter were found on neutrophil phagosomes (117). What’s more, chloride intracellular channel 1 (CLIC1) also contributes to the acidification and ROS production of macrophage phagosomes. CLIC5 on mitochondrial intima inhibits mitochondrial ROS production by acting on mitochondrial Complex II/III (118). Under pathological conditions, the dysfunction of chloride ion transport can lead to diseases. For instance, the cystic fibrosis transmembrane conductance regulator (CFTR) mutation has been widely reported to be an important cause of pulmonary cystic fibrosis (119).

**Figure 6:**
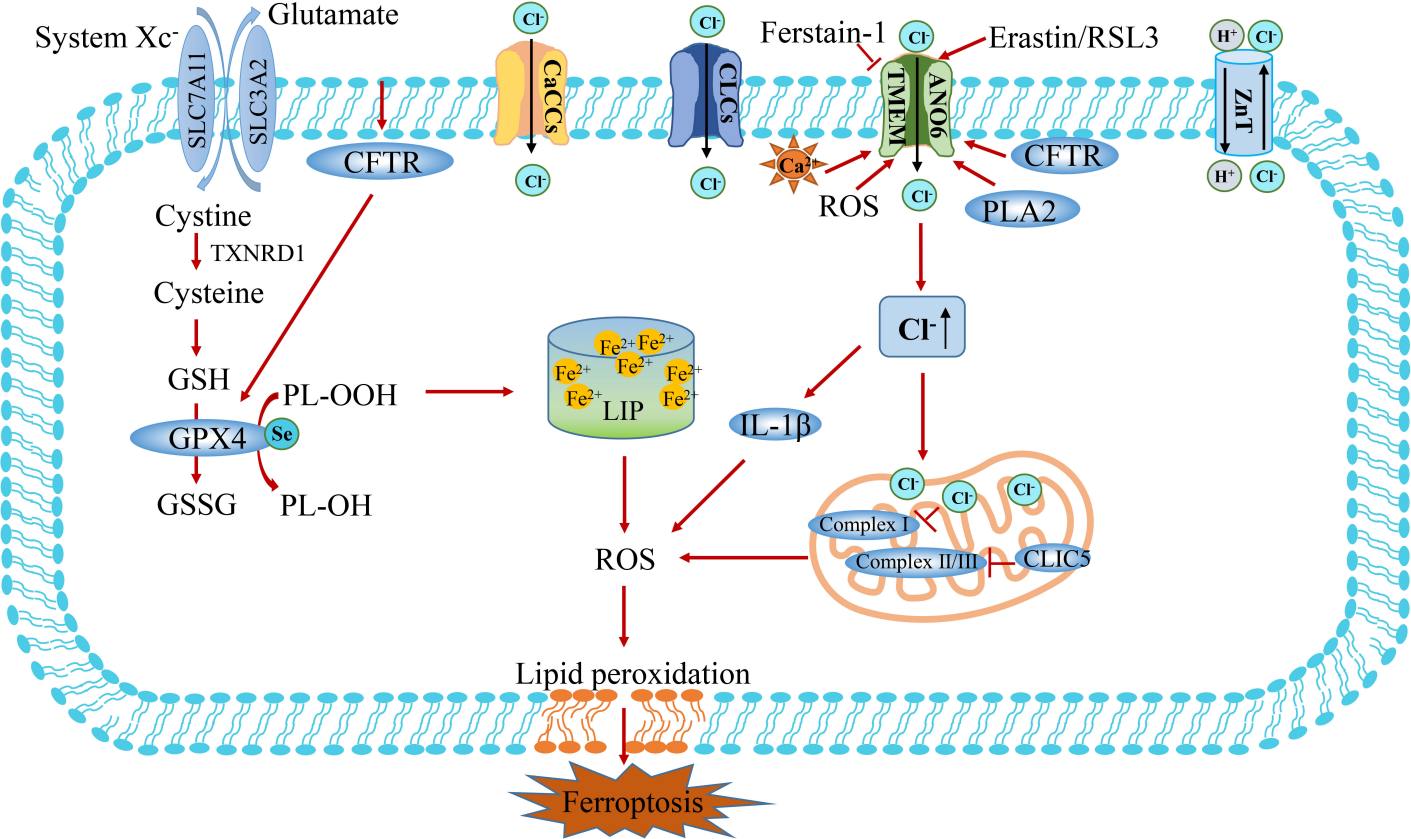
The crosstalk between Cl^-^ and Fe^2+^ metabolism. There are four types of chloride channels in eukaryotes, including voltage-gated chloride channels (CLCs), ligand-gated chloride channels, and cystic fibrosis transmembrane conductance regulatory channels (CFTR) and calcium-activated chloride channels (CaCCs). Calcium-activated chloride channel TMEM and intracellular chloride channel protein play a vital role in bridging Cl^-^ and ferroptosis. CLIC5 in mitochondrial intima inhibits the production of ROS by acting on mitochondrial complex II/III. TMEM16F (also known as ANO6) induces cell ferroptosis by regulating the flow of Cl^-^. Increased intracellular Cl^-^ concentration can act as a pro-inflammatory effector, induce the expression of IL-1β and production of ROS, reduce the activity of mitochondrial Complex I, and indirectly regulate the process of ferroptosis. CLIC5, chloride intracellular channel protein 5; TMEM16F, transmembrane protein 16F; PLA2, phospholipase A2.

In recent years, studies have reported that an imbalance of chloride ions and channels is associated with iron-dependent cell death (**Figure 6**) **(** 11, 120). TMEM16A-K (also known as Anoctamin 1-10) is a TMEM protein family consisting of 10 homologous proteins, belonging to calcium-activated chloride channels (121). TMEM16F (also known as ANO6) exposes phosphatidylserine to the cell surface by disrupting the phospholipids of the cell membrane, thereby activating a non-selective ionic current that destabilizes the plasma membrane and leads to cell death (122, 123). Further studies showed that TMEM16F is activated during ferroptosis, and the use of ferroptosis inhibitors Ferrostatin-1 or TMEM16F inhibitors can block chloride ion currents and inhibit cell death (**Figure 6**) **(** 11). The ionic current generated by TMEM16F depolarizes the cell membrane, causing the cell swelling. Due to cell swelling and phospholipid disorder, the plasma membrane is unstable lysis, which further explains the process of cell ferroptosis. Therefore, activation of TMEM16F may be a new strategy against cancer growth, although there is no small molecule activator targeting TMEM16F at present (11). In addition, increased intracellular Cl^-^ concentration exerts a pro-inflammatory effector, inducing the expression and secretion of IL-1β, induces the production of ROS, reduces the activity of mitochondrial Complex I, and indirectly regulates the process of ferroptosis (**Figure 6**) **(** 116).

In summary, it has been established that apart from iron ions participating in the ferroptosis process, other ions (such as Ca^2+^, Zn^2+^ and Cl^-^) also play an important role in ferroptosis. The crosstalk between ferrous and other ions in ferroptosis is summarized in **Table 1**. In particular, Ca^2+^-induced cell death is similar to Fe^2+^-dependent ferroptosis, which ferroptosis inhibitors can inhibit to a certain extent (55, 65). Zn^2+^ and Ca^2+^ share many transport and signaling pathways and can be used as novel intracellular signaling molecules (87, 88, 94). Therefore, we found that Ca^2+^ and Zn^2+^ may synergistically participate in Fe^2+^-induced oxidative stress and ferroptosis signal. Selenium is an important component of selenoproteins (such as GPX4). Selenoproteins exert biological functions in redox signaling and antioxidant defense, playing a regulatory role in ferroptosis. Cl^-^ is widely acknowledged as the most important anion. In recent years, it has been found that chlorine ion channel proteins are closely related to ferroptosis (11).

**Table 1:** The crosstalk between iron and other ions in ferroptosis.

Ions	Authors	Year	Target	Effects	Ref.
**Ca^2+^**	Sanmartin, CD et al.	2014	Iron	Iron induces RyR-mediated Ca^2+^ release	(124)
	Goldberg, J et al.	2020	Orail or Orai3	Knockdown of Orai1 or Orai3 protects against ferroptosis	(125)
	Chen, P et al.	2020	Ca^2+^/CaM	Ca^2+^ induces ROS accumulation and lipid peroxidation	(126)
	Angelova, PR et al.	2020	Calcium signaling	Ca^2+^ induces lipid peroxidation	(127)
	Inoue, M et al.	2021	NADPH	Ca^2+^ increases the activation of NADPH oxidase	(128)
	Henke, N et al.	2013	ORAI1 and GSH	Ca^2+^ entry through ORAI1 mediates cell death	(72)
	Xin, S et al.	2021	Lipid remodeling	Ca^2+^ promotes lipid elongation and desaturation	(129)
	Nakamura, T et al.	2021	MICU1	MICU1 increases lipid peroxidation	(130)
**Zn^2+^**	Zhang, C et al.	2020	ROS and iron	Zinc disrupts intracellular ROS and iron hemostasis	(8)
	Bishop, GM et al.	2007	ROS and GSH	Zinc stimulates the production of ROS and inhibits GSH	(90)
	Cortese, MM et al.	2008	Nrf2	Zinc increases the transcription of the Nrf2	(9)
	Arriaga, JM et al.	2014	MT1G	Zinc enhances the cytotoxicity of chemotherapy	(97)
	Knies, KA et al	2021	Mitochondrial	Zinc affects mitochondrial morphology	(101)
	Lin, LS et al.	2019	ROS	Zinc enhances oxidative stress-mediated cancer cell death	(131)
	Kilari et al.	2010	Iron uptake	Zinc induces iron uptake	(96)
	Slepchenko, KG et al.	2016	Nox and ROS	Zinc induces rapid ROS accumulation	(83)
	Noh, KM et al.	2010	Nox and PKC	Zinc influx activates PKC and produces ROS	(84)
	Walther, UI et al.	2003	GSSG	Zinc decreases GSSG reductase activity	(92)
	Puca, R et al.	2011	p53	Zinc increases the response of mutant p53 tumor cells	(132)
	Smith, A.F et al.	2012	HO-1	Zinc increases the expression of HO-1	(99)
	McMahon, M et al.	2010	Keap1 and NRF2	Zinc combined with Keap1	(100)
	Ahmad RS et al.	2019	ZIP14	Zinc upregulates ZIP14 expression	(103)
	Chen et al.	2021	ZIP7	ZIP7 triggered ER stresses	(133)
	Palmer, LD et al.	2019	GSH and NAD^+^	Zn induces GSH depletion and ferroptosis	(134)
	Li, H et al	2022	CircFOXP1	Zinc suppressed the cell viability	(135)
**Se**	Ingold, I et al.	2018	GPX4	Selenium inhibits GPX4 activity	(10)
	Yeo, JE et al.	2008	Mitochondrial	Selenium prevents mitochondria dysfunction	(136)
**Cl^-^**	Ousingsawat, J et al.	2019	TMEM16F/ANO6	Ca^2+^ activated cl^-^ channel contributes to ferroptosis	(11)
	Schreiber, R et al.	2018	PLA2 and ROS	TMEM16F/ANO6 are activated by PLA2 and ROS	(137)
	Valdivieso, ÁG et al.	2019	IL-1β and ROS	Cl^-^ increases ROS and decreases the activity of Complex I	(116)

## Ferroptosis and tumor therapy

Ferroptosis involves regulating cell death based on iron metabolism, which opens up new ideas and therapeutic strategies for tumor treatment. In addition, the crosstalk between ferrous ions and other ions is closely related, which may be gradually confirmed in future studies and provide a supplement for the treatment of ferroptosis-mediated tumors.

### Ferrous ions-induced ferroptosis in tumor therapy

Induction of ferroptosis can sensitize anti-tumor drugs and provide novel insights for improving the therapeutic effect of tumor treatment. Studies have substantiated that several drugs commonly used clinically, such as sorafenib, cisplatin, sulfadiazine, and artesunate, can induce ferrous-induced ferroptosis in various types of cancer (138–141). Moreover, it was found that changes in oncogene and tumor suppressor gene activity, such as Ras, Kras, p53 and BAP1, can regulate the expression of ferroptosis pathway proteins and mediate ferroptosis in tumor cells (20, 142–145). What’s more, immune checkpoint inhibitors have broadened the therapeutic landscape for patients with advanced tumors. Traditional immune-induced death methods include necrosis and apoptosis, but efficiency is observed in less than 1/3 of cases. Altering the mode of tumor cell death (e.g., ferroptosis) may increase the sensitivity of tumor immune killing (4, 146, 147). Similar studies have reported that combining immunotherapy and ferroptosis may have good stacking effects on tumor treatment sensitization and overcoming drug resistance, representing important perspectives for future tumor research. Finally, tumor cells often metastasize through the lymphatic system and the blood system. Interestingly, researchers found that intravenous injection of melanoma cells produced more metastatic tumors, and lymph node melanoma cells were more resistant to ferroptosis, suggesting that ferroptosis has an anti-tumor effect on lymph node metastasis (148).

It is widely thought that ferroptosis acts as a double-edged sword for tumor treatment, with the ability to promote or inhibit tumor death. Therefore, it is necessary to further explore therapeutic strategies for inducing ferroptosis in future studies, including screening the most optimal drugs, dosage, and interval of drug use, and clarifying indications for targeting ferroptosis to regulate tumor therapy.

### Other ions-induced ferroptosis in tumor therapy

Iron-induced cell death plays a central role in ferroptosis, but the mechanism of how the plasma membrane ruptured leads to the final cell death remains poorly understood. It has been reported that a sustained increase of cytoplasmic Ca^2+^ before cell rupture and death is another characteristic of ferroptosis, suggesting that Ca^2+^ plays an important role in the terminal event of ferroptosis (149). Chen et al. found that a natural product isolated from Dendrobium Chrysostom Lindl, can exert an anti-lu by inducing Ca^2+^/CaM-dependent ferroptosis (126). These results provide substantial evidence for the synergistic involvement of Fe^2+^ and Ca^2+^ in inducing ferroptosis. In a study by Plamena R et al. excessive α-synuclein in neurons could induce abnormal calcium influx signal, further inducing lipid peroxidation and ferroptosis (127). Xin et al. found that the MS4A15 protein located in the endoplasmic reticulum inhibits lipid elongation and desaturation by consuming Ca^2+^ stored in the endoplasmic reticulum lumen, thus protecting phospholipids from ferroptosis. Increasing intracavitary Ca^2+^ levels can be used to enhance the therapeutic sensitivity of refractory tumor cells, and manipulating Ca^2+^ homeostasis can balance lipid and cell death pathways, providing potential clinical applications for tumor therapy (129). Cell death induced by zinc ion toxicity is well confirmed, although recent studies reported that different zinc ion concentrations might mediate different death mechanisms. Palmer and colleagues found that nanogram zinc ion exposure can induce tumor ferroptosis, providing a theoretical basis for zinc as an anticancer drug in the treatment of non-small cell lung cancer (134). Chen demonstrated that although ferroptosis must depend on ferrous ion death, zinc is also an essential element for inducing breast cancer and kidney cancer cell death. Genome-wide RNAi screening identified SLC39A7 (also named ZIP7) as a novel determinant of ferroptosis by regulating zinc from the endoplasmic reticulum to the cytoplasm (133). In addition, zinc has been found to induce iron death in lung cancer by regulating circFOXP1 expression (135). Further studies substantiated that various zinc nanomaterials can destroy intracellular ROS and iron homeostasis, and thus induce ferroptosis, suggesting their potential for tumor treatment (8).

## Conclusions and perspectives

Ferroptosis is an iron-dependent cell death process characterized by excessive accumulation of reactive oxygen species and lipid peroxidation. As the mechanism of ferroptosis has been clarified, the comprehensive regulation based on iron and other ions metabolism brings potential clinical application value for anti-tumor therapy (4). Up to now, some researchers have tried to develop artificial ion transporters with anticancer activity for tumor treatment. Targeting ion channels and oxidative stress processes offers potential applications for diagnosing and treating of liver cancer and other tumors (150–152). Iron ions and the related mechanisms of ferroptosis have been established. However, there remain some unsolved problems in the mechanism of ferroptosis between iron and other ions, such as the essential difference and relationship between Ca^2+^ induced oxidative glutamate toxicity and cell death process and ferroptosis. What’s more, it remains unclear whether Fe^2+^ changes are accompanied by changes of concentration of other ions. Interestingly, Tsvetkov and Golub recently reported a novel type of cell death caused by Cu^2+^, which is called cuprotosis (3). They found that cells that rely on mitochondria for energy production are more sensitive to copper ionophores than cells treated with glucose. Whether there is a connection between cuprotosis and ferroptosis is worth of further investigation. Indeed, future studies should also investigate the specific role of ferroptosis involving different ions in diseases or tumors and develop new approaches to regulate ferroptosis.

## Author contributions

The review was designed and revised by WJ and HW. KK and LL conducted the literature collection and wrote the manuscript, which contributed equally to the manuscript. All authors contributed to the article and approved the submitted version.

## Funding

This work was supported by grants from scientific research fund of national health commission of China, key health science and technology program of Zhejiang Province (Grant No.WKJ-ZJ-2201); the “Pioneer” and “Leading Goose” R&D Program of Zhejiang (Grant No.2022C03099); Medical Health Science and Technology Project of Zhejiang Provincial Health Commission (Grant No.2022502425).

## Conflict of interest

The authors declare that the research was conducted in the absence of any commercial or financial relationships that could be construed as a potential conflict of interest.

## Publisher’s note

All claims expressed in this article are solely those of the authors and do not necessarily represent those of their affiliated organizations, or those of the publisher, the editors and the reviewers. Any product that may be evaluated in this article, or claim that may be made by its manufacturer, is not guaranteed or endorsed by the publisher.
